# What lessons can we learn? Clinical and epidemiological retrospective analysis of 267 patients with urticaria in a Brazilian tertiary center^☆^^☆☆^

**DOI:** 10.1016/j.abd.2020.12.003

**Published:** 2021-05-24

**Authors:** Roberta Fachini Jardim Criado, Paulo Ricardo Criado, Nathalia Baldavira, Debora Cardial, Ana Maria Gimenez-Arnau, Carlos D’Apparecida Machado Filho

**Affiliations:** a Department of Dermatology, Centro Universitário Saúde ABC, Santo André, SP, Brazil; b Dermatology Department, Hospital Del Mar Imim, Barcelona, Spain

**Keywords:** Angioedema, Biomarkers, pharmacological, Epidemiology, Treatment outcome, Urticaria

## Abstract

**Background:**

There are few epidemiological studies of urticaria, published in the indexed literature (PubMed/Medline).

**Objective:**

The study aimed to evaluate the epidemiological and clinical data among patients with urticaria/angioedema attending a reference clinic in Brazil.

**Methods:**

Two hundred sixty-seven patients were evaluated retrospectively considering demographic data, time course of the disease, triggering symptoms, the presence of angioedema, complementary laboratory tests including total blood count, reactive-C protein, erythrocyte sedimentation rate, IgE serum levels, and other, as necessary.

**Results:**

The most commonly diagnosed type of urticaria was chronic spontaneous urticaria (56.93%). Angioedema was associated with chronic urticaria in 108 patients (40.08%).

**Study limitations:**

Unicentered and retrospective.

**Conclusion:**

Some relevant findings in this study are the observation of a female prevalence of cases (4-females: 1-man), a result more elevated than demonstrated in previous studies in Europe and Asia, the median age was 43-years old and the delay of time between the diagnosis of urticaria and the admission for treatment in a specialized center was approximately 2-years. Other multicenter studies can better establish these differences in Brazilian patients.

## Introduction

Urticaria can be classified as acute, if it lasts less than 6-weeks, or chronic if it lasts more than 6-weeks.^1^ The diagnosis is mainly clinical, and most urticaria cases are considered spontaneous.^1^ Because it is an underdiagnosed disease, it is very difficult to find accurate epidemiological data.^2^ It is believed that 15% to 20% of people will present at least one episode of urticaria during their lives, with a greater incidence in patients between 20 and 40 years old.^3^, ^4^ It is estimated that between 0.11% to 1% of the urticaria cases are chronic urticaria.^5^

In the Brazilian population, there are few epidemiological studies in urticaria conducted in the last decade, retrospectively or prospectively, published in the indexed literature (PubMed/Medline).^4^, ^6^, ^7^

The authors described the profile of 267 patients registered in the Urticaria Unit of Dermatology Department during the period from January 2011 to December 2017. This study was approved by the local Ethical Committee.

## Methods

A retrospective study was performed based on the evaluation of the medical registration of 336 patients of the Urticaria Unit of the Department of Dermatology of Faculdade de Medicina do ABC. Unfortunately, 69 patients were excluded from the study due to incomplete or uncollected exams during their follow-up. The authors collected epidemiological information such as age, gender, time of follow-up, time to seek for medical evaluation, and clinical data as type of urticaria (acute or chronic, spontaneous or induced), laboratory exams including blood cells counts (erythrocytes, leukocytes, neutrophils, basophils, eosinophils, platelets, hemoglobin), C-reactive protein (CRP; serum CRP concentration was measured by the nephelometric method and elevated serum CRP was defined as ≥6.0 mg/L), Erythrocyte Sedimentation Rate (ESR) and total IgE serum levels (kU/L; it was determined by the commercial ImmunoCAP250 system, Pharmacia™, Uppsala, Sweden, normal value <100 kU/L). Other procedures were performed if indicated by the clinical setting, such as the Autologous Serum Skin Test (ASST) if there was a familial or personal history of autoimmune thyroid disease, following the technical recommendations of Konstantinou et al.,^8^ and cutaneous biopsies in cases suspected of urticarial vasculitis. For all patients that during anamnesis, were suspected of induced-urticarias, the anti-H1 treatment was stopped, and the specific tests for chronic induced-urticaria were applied as recommended by Magerl et al.^9^

### Data collection

The data for the patients enrolled in the Urticaria Unit of FMABC was collected from the medical charts. The authors have included all the patients that had the information required as described above. Patients that did not have the age, gender, time to seek medical care, time of follow-up for the urticaria, presence or absence of angioedema, and the laboratory exams were excluded from this study. The study excluded all cases of Angioedema without urticaria suspicious of hereditary or acquired bradykinin angioedema.

The limitations of this study include the retrospective design, a tertiary medical center population, and low statistical power due to the small sample size.

For the analysis of the data collected, it was established a statistical significance of 95% (p < 0.05). The qualitative values were analyzed by their mean and relative value. The quantitative data were tested for their normality in the Shapiro-Wilk test. For p < 0.05, the distribution was considered non-normal, and it was analyzed by the interquartile range and median. For p > 0.05, the data was analyzed by the mean and standard deviation. The program Stata 11.0 was used.

## Results

The prevalence of urticaria in the dermatology department was 1.71% from January 2011 to March 2017 (336 urticaria patients diagnosed during this period, from a total of 19.568 outpatient care patients during the same period in the Dermatology Department for all dermatological care). The gender of most of the patients was female (214 women, 80.15%). The age of the youngest patient was 8-years old, and the oldest, 83-years old (median 43-years old) (IR: 33–54), and most patients (59.93%) were in the 3^rd^, 4^th^ and 5^th^ decades (Table 1). The average medical follow-up was 30.26 months (IR*: 5–41.5), and the mean value of time to be referred for the first specialized medical consultation was 24-months (IR*: 6–48).

**Table 1:** Demographic data, types of urticaria, presence of angioedema, complementary exams, and time of course of the diseases on 267 patients included in this study.

**Gender**		Female 214 (80.15%)			
		Male 53 (19.85%)			
**Age in years (Range**^a^**)**	Number of patients (%)	Mean		IR^b^	
	0–10 (10; 3.74%)				
	11–20 (18; 6.74%)				
	21–30 (34;12.74%)				
	31–40 (42; 15.74%)				
	41–50 (72; 26.97%)	43.6		33–54	
	51–60 (46; 17.22%)				
	61–70 (27;10.11%)				
	71–80 (17; 6.37%)				
	>80 (0.37%)				
**Types of urticaria: number of patients (%)**	Acute	6 (2.24%)			
	Chronic	Spontaneous		152 (56.93%)	
		Induced		96 (35.95%)	
	Urticaria vasculitis	11 (4,13%)			
	Autoinflammatory diseases	2 (0.75%)			
**Angioedema: number of patients (%)**		108 (40.44%)			
**Autologous serum skin test (number of tests performed)**		115 (43.07%) – positive in 80 patients (69.56%)			
**Cutaneous biopsy (number of patients were performed)**		42 (15.73%)			
**Complementary blood exams**		Blood cells count	Exam	Mean	SD
			Hemoglobin	14.04	2.04
			Hematocrit	41.23	5.13
		Total IgE serum levels (kU/L)		262.05	42.49
		ESR		22.1	15.75
**Complementary blood exams with elevated results**		Hemoglobin		18 (6.74%)	
		Hematocrit		27 (10.11%)	
		CRP Value		35 (13.1%)	
		ESR		110 (41.19%)	
		Total IgE serum levels		86 (32.2%)	
**Time of urticaria history referred for patients before the first consultation, number of patients (%)**		Until 1-year		123 (46.11%)	
		From 1 to 5 years		104 (38.89%)	
		From 5 to 20 years		40 (15%)	

CRP, C-reactive Protein; ESR, Erythrocyte Sedimentation Rate.

Demographic data, types of urticaria, presence of angioedema, exams, and time of course of the diseases.

a Non-normal distribution.

b Interquartile Range.

Chronic urticaria was the most diagnosed type of urticaria (spontaneous 56.93% and 35.95% of induced-urticaria). Eighty-two patients (33.06%) with chronic urticaria presented both spontaneous induced-urticaria. Among chronic induced-urticaria patients, 50% of the cases were classified as symptomatic dermographism (48 patients), 22.91% delayed-pressure urticaria (22 patients), 4.16% solar urticaria (4 patients), 15.62% as cholinergic urticaria (15 patients), and 7 patients presented symptomatic dermographism and delayed pressure urticaria (7.29%). One hundred and eight patients with chronic spontaneous urticaria (CSU) - chronic inducible urticaria (CINDU) (40.44%) had angioedema.

Only 6 patients registered showed acute spontaneous urticaria (2.24%). Eleven (4.13%) patients were diagnosed with urticarial vasculitis after cutaneous biopsies, and in most of these cases, nine patients did not show hyperchromic residual lesions or purpura on the description of the physical exam. Two cases (0.75%) of acquired autoinflammatory diseases were diagnosed due to recurrent fever episodes, arthralgia, urticarial lesions, and persistent laboratory exams suggesting systemic inflammation, and these cases were classified as Adult-Onset Still Disease (AOSD) and the other as Neutrophilic Urticarial with Systemic Inflammation (NUSI), as previously described by Mehrpoor et al. and Belani et al. and referred to the Rheumatology Department of the Institution (Table 1).^10^, ^11^ These patients were excluded from the analysis of CU.

Most of the patients had normal blood cells counts and CRP. In the blood cell count, the mean of hemoglobin was 14.04 (SD = 2.04), and hematocrit test 41.23 (SD = 5.13). The mean of IgE was 262.05 (SD = 424.9). The IgE was elevated in 82 patients (32.2%), and distributed under the following ranges, 101–150 kU/L (41 patients, 50%), 151–500 kU/L (23 patients, 28%), and ≥ 500 kU/L (18 patients, 22%), but there were no significant differences between the patients with isolated CSU and CSU-CINDU. The Erythrocyte Sedimentation Rate (ESR) mean was 22.1 (SD = 15.75), therefore, abnormal in 41.13% of the patients (normal range in males is ≤15 mm/hour and ≤ 20 mm/hour in female patients), and CRP was elevated (normal range ≤5 mg/dL) in 35 patients (13.1%) (Table 1).

Results of Hemoglobin (Hb) and Hematocrit (Hct) are demonstrated in Table 1. Regarding the urticaria duration (time of evolution of the disease), 123 patients had their disease for one year (46.11%), 104 (38.89%) for 1 to 5 years, and 40 patients (15%) through 5 to 20 years (Table 1).

## Discussion

The prevalence of urticaria in the dermatology department was 1.71% during the seven years of this retrospective study. Balp et al.,^2^ in 2017 published a retrospective, cross-sectional study, pooled data from 2011, 2012, and 2015 National Health and Wellness Survey in Brazil (n = 36,000). They found in their study that the prevalence of CU was 0.41% (n = 249 patients) among respondents (aged >18 years) diagnosed with and treated for chronic urticaria.^2^ However, these authors collected data not only from tertiary centers, as the Urticaria Unit in this study. In another tertiary Hospital in Brazil, the data published in 2017 showed an incidence of 1.9% new cases of urticaria/angioedema during the year 2014, demonstrating results that were similar to this study.^12^

In the present study, the mean age of patients with urticarial was 43 years old. Dias et al. studied the impact of Chronic Urticaria (CU) on quality of life in a Brazilian University hospital in 2016.^6^ The authors studied 112 patients with CU and found a demographic profile comparable to the present study. Dias et al.^6^ in their transversal cohort study, described that 85.72% of the CU patients were female, with a mean age of 46-years (18–90), a prevalence in concordance to this study (80.15% of female patients and a mean age of 43-years old). In another Brazilian study, in a tertiary University hospital, Cordeiro et al.^7^ studied autoimmunity in 254 patients with CU/angioedema and found a prevalence of 79.5% of female patients and a range of age between 13–80 years old. All four Brazilian studies, including this present study, on demographic data on urticaria in Brazil, showed results in opposition to most studies of demographics of urticaria in Asia and Europe.

Maurer et al.^13^ in Europe emphasized that most studies show that women suffer from urticaria nearly twice as often as men do. This is not only true for chronic spontaneous urticaria but also urticaria in general.^13^ Neither the criteria for selection nor the observed country or time of the distinct studies conducted in Europe seems to alter this clinical scenario.^13^ In Brazil, the studies suggested twice the incidence of CU in female in comparison to Europe. In a European review about CSU, Maurer et al.^13^ presented an statistical analysis of patients presenting with non-acute urticaria suggesting 66%–93% have chronic spontaneous urticaria, 4%–33% physical urticaria, and 1%–7% cholinergic urticaria. Our data is remarkably similar to these previous results in Europe; in the patients studied, the authors found CU was the most commonly diagnosed type (CSU 56.93% and 35.95% of chronic induced-urticaria, CINDU). However, in our patients, 15.62% were diagnosed with cholinergic urticaria, a higher prevalence than in the European data.^13^ Possibly, the elevated climate temperature during almost all months of the year in Brazil could explain this prevalence of cholinergic urticaria in patients living in this country.

Also, in a review of physical urticaria, Trevisonno et al.^14^ conducted a systemic review and meta-analysis of ten studies to determine the prevalence of physical urticaria in chronic urticaria patients. Sample sizes ranged from 202 to 4157 patients. The estimate of the combined prevalence of physical urticaria including and excluding cholinergic forms in all cases of chronic urticaria was 13.1% and 14.9%, respectively. These results suggest that cholinergic urticaria is an important subset of chronic urticaria and the clinicians should be aware of this important condition to effectively manage patients. Additionally, Trevisonno et al.^14^ emphasized in their conclusion that Physical Urticarias (PU) affect 6% to 30% of all patients with CU. The author’s results showed a high prevalence, but among our 35.95% of patients with CINDU, this study included cases of cholinergic urticaria (15.62%), which turn CINDU more prevalent in the present study.

Kolkhir et al.^15^ studied retrospectively data from 1,253 CSU patients from the urticaria specialty clinics of the dermatological departments of Charité – Universitätsmedizin Berlin, Germany (n = 1,023) and I.M. Sechenov First Moscow State Medical University, Russia (n = 230). The authors found CRP elevated in one-third of patients with CSU.^15^ However, in the 267 studied patients, CRP was elevated in only 13.1% of the cases. ESR is another exam that is a well-known non-specific marker of inflammation.^15^, ^16^ In contrast to ESR, CRP is not or is less likely to be affected by other factors such as size, shape, and number of red blood cells, female sex, and pregnancy as is ESR.^15^, ^16^ In this present study, 41.13% of patients showed elevated levels of ESR, and this result could be related to a greater number of female patients in the cohort studied. The patients included in Kolkhir et al. study showed ESR elevated in 18.9% of patients, however, in this study, the gender prevalence was 3 females:1 male, in opposition to a prevalence of 4 female:1 male in our patients. As discussed above, the female gender can affect ESR levels.^15^, ^16^

Total IgE serum levels were elevated in 86 of our patients (32.2%) with a mean of 262.05 kU/L (± SD 42.49) in all patients with urticaria. Chang et al. in 2013, studied acute and chronic urticaria in a total of 165 patients (104 with acute and 61 with chronic urticaria) in Taiwan.^17^ The authors found a geometric mean of 94.25 kU/L (± SD 4.38) total IgE serum levels in 100 patients with acute urticaria, and in 60 patients with chronic urticaria a geometric mean of 159.58 kU/L (± SD 4.43), in concordance with the author’s results. Otherwise, Cordeiro et al., in Brazil, studying 254 patients with chronic urticaria/angioedema found 60% of their patients with total IgE > 100 kU/L.^7^ Martins et al.^4^ in Brazil (2018) in a retrospective study conducted on a tertiary University Hospital enrolled 97 patients with CSU and observed total serum IgE levels elevated in 22 of 39 patients (56.41%).

In 2018, Eun et al published an interesting study about the natural course of new-onset urticaria, as a result of a 10-year follow-up, nationwide, population-based study in South Korea.^18^ The national sample cohort data of their study consisted of 1,027,260 subjects with no history of urticaria during the last two years (2002–2003). From 2004 to 2013, a total of 49,129 patients with new-onset urticaria were identified.^18^ The 10-year cumulative incidence rate of urticaria for the general population was 4.9% and that of chronic urticaria among patients with new-onset urticaria was 7.8%.^18^ Remission rates of chronic urticaria were 52.6% at one year and 88.9% at 5-years.^18^ In this center, the course of urticaria showed 123 patients had up to one year of disease (46.11%), 104 patients from 1 to 5 years of disease (38.89%), and 40 patients had a long course of urticaria, from 5 to 20 years (15%). Despite methodological and populational differences between the present study and those of the South Korean authors, the percentages of remissions in patients with urticaria overall were remarkably similar.^18^

It is undeniable that women are more likely to have urticaria than men.^18^ Eun et al.^18^ showed a clear female predominance of new-onset urticaria only for those aged between 20–44 and 45–64 years.^18^ The authors suggested that female predominance of urticaria in specific age groups could be due to estrogen.^18^ It is postulated that estrogen enhances humoral immunity and antibody synthesis.^18^ Experimental data have shown that estradiol can enhance mast cell activation and allergic sensitization in rodent models.^18^ In the present study, the female prevalence was unequivocal (4 females:1 male). Beyond estrogen involvement in urticaria pathogenesis, a social aspect can exert influence in the cohort of patients: women look for medical assistance more frequently than men. In Spain, in a retrospective study which enrolled 549 patients with CSU, 402 (73%) were female, gender data close to our results.^19^

In the present study, the mean follow-up time was approximately two and a half years, and the time the disease lasted at the Institution was from 1 to 20 years showing that CU is a long lasting disease.

The diagnosis of urticaria is mainly clinical; only rarely auxiliary exams or skin tests are performed to clarify a possible etiology, such as Autologous Serum Skin Test (ASST). ASST is correlated to disease severity by some authors.^15^ The authors performed the ASST in 115 patients (43.07%), and these tests were positive in 80 patients (69.56%). Kolkhir et al.^15^ in Moscow found a positive ASST in 61.4% (78/127) of their patients. Curto-Barredo et al.^19^ studied retrospectively a large sample of 549 patients with CSU, during a period from 2001 to 2014 and performed ASST in 64.5% of their patients, with a positive ASST in 192 (54.2%), a similar result obtained in our patients.

Regarding the biopsy in the urticarial lesions in the patients, it was performed only because of suspicion of Urticaria Vasculitis (UV), chronic urticaria refractory to conventional therapy, and when the diagnosis of urticaria pigmentosa was suspected. This explains why only 42 patients (15.73%) had the results of the biopsy in their medical chart and it led to a diagnosis of urticarial vasculitis in eleven patients (4.13% of the 267 total urticaria patients). Usually urticaria is not complicated and is susceptible to conventional treatment.^4^ The prevalence of UV is 5 to 10 percent in patients with chronic urticaria, results also found in the present study, and less than five percent in patients with Hypocomplementemic Urticaria Vasculitis Syndrome (HUVS).^20^, ^21^

This retrospective study of 7-years in an Urticaria Unit of a tertiary center in Brazil showed limitations proper to this kind of study design (retrospective), where more detailed data often is missing. However, the authors are adding new data to the Latin American profile of patients suffering from urticaria in the last decade.

## Conclusion

The study demonstrated that there is a clear predominance of the female gender with urticaria, more than is observed in Europe and some countries of Asia. Most urticaria patients in the outpatient clinic are middle-aged women, with chronic urticaria without angioedema, and a few alterations in their laboratory exams, with almost one-third of patients, having IgE and ESR serum levels elevated. The median time to obtain a specialized consultation in an Urticaria Reference Center was 24-months, and most of the patients were followed in the Urticaria Unit for a period of 30-months. More efforts are needed to recognize the disease and to obtain medical assistance for urticaria patients in specialized units.

## Financial support

This study was funded in part by Coordenação de Aperfeiçoamento de Pessoal de Nível Superior – Brasil (10.13039/501100002322CAPES) – Código Financeiro 001.

## Authors’ contributions

Roberta Fachini Jardim Criado: Design and planning of the study; drafting and editing of the manuscript; collection, analysis, and interpretation of data; effective participation in research orientation; intellectual participation in the propaedeutic and/or therapeutic conduct of the studied cases; critical review of the literature.

Paulo Ricardo Criado: Approval of the final version of the manuscript; design and planning of the study; drafting and editing of the manuscript; effective participation in research orientation; critical review of the literature; critical review of the manuscript.

Nathalia Baldavira: Design and planning of the study; drafting and editing of the manuscript; collection, analysis, and interpretation of data.

Debora Cardial: Design and planning of the study; drafting and editing of the manuscript; collection, analysis, and interpretation of data.

Ana Maria Gimenez-Arnau: Effective participation in research orientation; critical review of the literature; critical review of the manuscript.

Carlos D'Apparecida Machado Filho: Effective participation in research orientation; intellectual participation in the propaedeutic and/or therapeutic conduct of the studied cases; critical review of the literature.

## Conflicts of interest

RFC and PRC are medical consultants to Novartis and Takeda. AMG-A is a medical consultant to Uriach Pharma, Genentech and Novartis. She is the recipient of research grants from Intendis-Bayer, Uriach Pharma and Novartis, and participated in educational activities sponsored by Uriach Pharma, Novartis, Genentech, Menarini, Glaxo Smith & Kline, Merck MSD, Almirall and Leo Pharma.
